# Identification of miRNAs as the Crosstalk in the Interaction between Neural Stem/Progenitor Cells and Endothelial Cells

**DOI:** 10.1155/2020/6630659

**Published:** 2020-12-11

**Authors:** Xin Wang, Simin Li, Yihong Ma, Yuzhen Xu, Anthony Chukwunonso Ogbuehi, Xianda Hu, Aneesha Acharya, Rainer Haak, Dirk Ziebolz, Gerhard Schmalz, Hanluo Li, Sebastian Gaus, Bernd Lethaus, Vuk Savkovic, Zhiqiang Su

**Affiliations:** ^1^Department of Neurology, First Affiliated Hospital of Harbin Medical University, Harbin 150001, China; ^2^Department of Cariology, Endodontology and Periodontology, University Leipzig, Liebigstr. 12, 04103 Leipzig, Germany; ^3^Department of Neurology, Graduate School of Medical Sciences, Faculty of Life Sciences, Kumamoto University, Kumamoto, Japan; ^4^Department of Neurology, Shanghai Tenth People's Hospital, Tongji University School of Medicine, No. 301 Middle Yanchang Road, Shanghai, China; ^5^Faculty of Physics, University of Münster, Wilhelm-Klemm-Straße 9, 48149 Münster, Germany; ^6^Laboratory of Molecular Cell Biology, Beijing Tibetan Hospital, China Tibetology Research Center, 218 Anwaixiaoguanbeili Street, Chaoyang, Beijing 100029, China; ^7^Faculty of Dentistry, University of Hong Kong, Hong Kong, China; ^8^Department of Cranio Maxillofacial Surgery, University Clinic Leipzig, Liebigstr. 12, 04103 Leipzig, Germany

## Abstract

**Aim:**

This study is aimed at identifying genetic and epigenetic crosstalk molecules and their target drugs involved in the interaction between neural stem/progenitor cells (NSPCs) and endothelial cells (ECs).

**Materials and Methods:**

Datasets pertaining to reciprocal mRNA and noncoding RNA changes induced by the interaction between NSPCs and ECs were obtained from the GEO database. Differential expression analysis (DEA) was applied to identify NSPC-induced EC alterations by comparing the expression profiles between monoculture of ECs and ECs grown in EC/NSPC cocultures. DEA was also utilized to identify EC-induced NSPC alterations by comparing the expression profiles between monoculture of NSPCs and NSPCs grown in EC/NSPC cocultures. The DEGs and DEmiRNAs shared by NSPC-induced EC alterations and EC-induced NSPC alterations were then identified. Furthermore, miRNA crosstalk analysis and functional enrichment analysis were performed, and the relationship between DEmiRNAs and small molecular drug targets/environment chemical compounds was investigated.

**Results:**

One dataset (GSE29759) was included and analyzed in this study. Six genes (i.e., MMP14, TIMP3, LOXL1, CCK, SMAD6, and HSPA2), three miRNAs (i.e., miR-210, miR-230a, and miR-23b), and three pathways (i.e., Akt, ERK1/2, and BMPs) were identified as crosstalk molecules. Six small molecular drugs (i.e., deptropine, fluphenazine, lycorine, quinostatin, resveratrol, and thiamazole) and seven environmental chemical compounds (i.e., folic acid, dexamethasone, choline, doxorubicin, thalidomide, bisphenol A, and titanium dioxide) were identified to be potential target drugs of the identified DEmiRNAs.

**Conclusion:**

To conclude, three miRNAs (i.e., miR-210, miR-230a, and miR-23b) were identified to be crosstalks linking the interaction between ECs and NSPCs by implicating in both angiogenesis and neurogenesis. These crosstalk molecules might provide a basis for devising novel strategies for fabricating neurovascular models in stem cell tissue engineering.

## 1. Introduction

It is well known that the neurovascular unit (NVU) comprises a collection of cells (e.g., endothelial, neural, and glial cells), which can control interactions between neurons and the vasculature. Based on the concept of NVU, the construction of 3D neurovascular tissue has emerged as a promising approach in the field of tissue engineering [1, 2]. Constructing 3D neurovascular tissues by combining neurogenesis and angiogenesis models is viewed as a potentially effective strategy to facilitate functional recovery in ischemic stroke [3]. Increasing evidence has shown that the cell contact-dependent interactions between neural stem/progenitor cells (NSPCs) and endothelial cells (ECs) can drive the coupling of neurogenesis and angiogenesis. On the one hand, NSPCs can promote morphogenesis and angiogenesis of ECs by expressing angiogenic factors such as vascular endothelial growth factor (VEGF) [4]. In turn, VEGF has been demonstrated to promote neurogenesis, neuronal patterning, neuroprotection, and glial growth of NSPCs [5]. On the other hand, ECs can also stimulate survival, proliferation, neuronal differentiation, and neurogenesis of NSPCs by secreting neurotrophic factors such as brain-derived neurotrophic factor (BDNF) [6–8]. Conversely, BDNF has been shown to support the cell-cell contacts and survival of ECs, suggesting that it plays a critical role in maintaining vessel stabilization and mediating angiogenesis [9]. Overall, current evidence indicates that the interactions between NSPCs and ECs are governed by several common crosstalk factors that regulate both the neurogenic and angiogenic processes.

A previous study using microarray analysis investigated the molecular mechanisms underlying the interaction between NSPCs and ECs at the mRNA and miRNA levels [10]. This study focused on investigating the NSPC-induced molecular alterations in the angiogenesis of ECs and the EC-induced molecular alterations in the neurogenesis of NSPCs. However, this study did not explore the genetic and epigenetic crosstalks underlying the reciprocal regulations between NSPCs and ECs from a systemic and comprehensive perspective. Bioinformatics techniques can be used to analyze and interpret data obtained by microarray datasets in order to identify the crosstalk between NSPCs and ECs at the gene and microRNA (miRNA) levels. Since targeting miRNAs with small molecules and environmental chemical compounds is a promising therapeutic strategy for human diseases [11, 12], it is necessary to identify the molecules and compounds that might be utilized in targeted drug delivery to construct neurovascular tissue. To the authors' knowledge, this is the first report using bioinformatics techniques to identify the crosstalk genes, signaling pathways, and miRNAs, as well as the crosstalk miRNA targeting drugs that are involved in the reciprocal regulation between NSPCs and ECs. As crosstalk molecules and their related drugs identified in this study are implicated in regulating neurogenic and angiogenic processes, their identification can potentially enable the development of novel strategies for fabricating neurovascular models using 3D tissue engineering.

## 2. Materials and Methods

### 2.1. Procurement of Microarray Datasets

A single dataset (GSE29759) using the Mus musculus model was retrieved from the Gene Expression Omnibus (GEO) database [10]. This dataset investigated both the mRNA and miRNA expression profiling. The study design of this dataset was established with four groups: monoculture of ECs (group A), ECs grown in EC/NSPC cocultures (group B), monoculture of NSPCs (group C), and NSPCs grown in EC/NSPC cocultures (group D).

These four groups were divided into two categories. First, the NSPC-induced EC alterations were analyzed by comparing expression profiles between groups A and B. In the comparison between groups A and B, group A was considered the control group, while group B was regarded as the experimental group. Second, the EC-induced NSPC changes were analyzed by comparing groups C and D. Likewise, in the comparison between groups C and D, group C was considered the control group, while group D was regarded as the experimental group.

### 2.2. Differential Expression Analysis

Differential expression analysis was performed using the R package “limma” [13] for identifying differentially expressed genes (DEGs) and differentially expressed miRNAs (DEmiRNAs) relevant to EC-induced NSPC changes and NSPC-induced EC changes, respectively. Genes and miRNAs with *P* value < 0.05 and ∣log2FC | ≥0.5 were considered DEGs and DEmiRNAs. Overlapping DEGs and DEmiRNAs between these two categories were determined using Venn diagrams.

### 2.3. Functional Enrichment Analysis of DEGs

Functional enrichment analysis of the overlapping DEGs was based on Gene Ontology (GO) terms, especially biological process (BP), as well as KEGG (Kyoto Encyclopedia of Genes and Genomes) pathways. This analysis was conducted by using the clusterProfiler package in the R program (significance level *P* < 0.05) [14].

### 2.4. The Construction of a PPI Network

Protein-protein interaction (PPI) pairs corresponding to DEGs were obtained using the STRING database [15]. Protein-protein interaction (PPI) networks of NSPC-induced EC changes (EC-PPI) and EC-induced NSPC changes (NSPC-PPI) were constructed with the “Cytoscape” software platform [16]. The topological characteristics of each PPI network were determined using the “NetworkAnalyzer” tool. A Venn diagram was used to identify the overlapping DEGs between the EC-PPI network and the NSPC-PPI network.

### 2.5. Identification of Transcription Factors-Target DEGs

To identify regulatory transcription factors (TFs) that control the DEGs at a transcriptional level, TF-target DEG interactions were obtained using the Transcriptional Regulatory Relationships Unraveled by Sentence-based Text mining (TRRUST) database Version 2 [17]. The TF-target DEG interaction networks, respectively, for the DEGs involved in the EC-induced NSPC alteration and NSPC-induced EC alteration, were constructed based on these TF-target DEG interaction pairs. The topological characteristics (e.g., degree and betweenness centrality) of the nodes in the networks were also calculated.

### 2.6. Prediction of miRNA Targets

The experimentally validated gene targets of DEmiRNAs were obtained by three databases, including miRTarBase [18], miRecords [19], and TarBase v7 [20]. The computationally predicted gene targets of DEmiRNAs were obtained from eight databases including TargetScan [21], starBase [22], miRNAMap [23], miRDB [24], miRWalk [25], RNAhybrid [26], and miRanda [24]. Combining the experimentally validated and computationally predicted gene targets, a set of gene targets of the DEmiRNAs was obtained. From these, differentially expressed gene targets were identified and termed as miRNA-DEtarget. The DEmiRNA-target network involved in the EC-induced NSPC alteration and NSPC-induced EC alteration was constructed, respectively, using the clusterProfiler package in the R program [14]. The topological characteristics of nodes in this network were also calculated.

The DEmiRNA-DEtargets for each category were subjected to functional enrichment analysis in order to identify the signaling pathways with significant involvement. Corresponding miR-DEG-pathway interaction networks for each miRNA were constructed to examine DEtarget genes and their relevant enriched functional pathways.

### 2.7. The Relationship between DEmiRNAs and Small Molecular Drugs

The DEmiRNAs determined using the mouse genome were assigned corresponding Homo sapiens gene identities and further converted to the names of probes based on the HG-U133A platform. The human probe identities were uploaded to the Cmap web tool [27], and small molecular signatures of the selected DEmiRNAs were calculated by the following three formulas:

(1) $$\begin{array}{c} a=\overset{t}{\underset{j=1}{\mathrm{Max}}}\left[\frac{j}{t}-\frac{V\left(j\right)}{N}\right],\\ b=\overset{t}{\underset{j=1}{\mathrm{Max}}}\left[\frac{V\left(j\right)}{N}-\frac{\left(j-1\right)}{t}\right],\\ {\text{KS}}_{\text{up}/\text{down}}=\left\{{}_{-b\;\left(b>a\right)}^{a\;\left(a>b\right)}\right\}. \end{array}$$

The variable *t* represents the number of DEmiRNAs which could be either upregulated or downregulated; *j* represents the *j*th gene based on the rank of the differential expression; *N* denotes the magnitude of the ranked small molecular signature; the element *V*(*j*) of a vector *V* is the position of the *j*th target gene in the ordered small molecular signature. The corresponding KS_up_ and KS_down_ generated were integrated into the *S* score as follows: *S* is equal to 0 when KS_up_ and KS_down_ have the same sign and is equal to KS_up_ − KS_down_ otherwise.

### 2.8. Predicting the Relationship between DEmiRNAs and Environmental Chemical Compounds

The relationship between environmental chemical compounds and DEmiRNAs was determined by downloading the corresponding interaction pairs from the Comparative Toxicogenomics Database (CTD) [28]. Highly correlated DEmiRNAs-environmental chemical compounds were then determined by filtering using a hypergeometric test. The formula of the hypergeometric test is as follows:

(2) $$\begin{array}{c} P\left(k,N,M,n\right)=\frac{\left(\begin{array}{c} M\\ k \end{array}\right)*\left(\begin{array}{c} N-M\\ n-k \end{array}\right)}{\left(\begin{array}{c} N\\ n \end{array}\right)},\;k=0,1,2,3,\cdots ,M. \end{array}$$

*N* represents the total number of DEmiRNAs; *n* represents the number of DEmiRNAs corresponding to a specific chemical compound; *M* represents the number of the target genes of a specific miRNA; *k* represents the genes which not only belong to the target genes of DEmiRNAs but also are related to chemical compounds.

### 2.9. Crosstalk miRNA Analysis

The crosstalk miRNAs were explored using the “Meet/Min” score based on the gene targets, small molecules (DEmiRNA-molecule interaction pairs), environmental chemical compounds, signaling pathways, and biological processes in GO terms. The “Meet/Min” score was calculated as follows:

(3) $$\begin{array}{c} \text{Score}\left(i,j\right)=\frac{\left|\text{targets}\left(i\right)\cap \text{targets}\left(j\right)\right|}{\min\left(\;|\;\text{targets}\left(i\right)\;|\;,\text{targets}\left(j\right)\;|\;\right)\;}. \end{array}$$

For one miRNA pair, *i* and *j*, their differentially expressed target gene sets were targets(*i*) and targets(*j*), respectively.

The number of common differentially expressed targets of the two miRNAs was divided by the size of the smaller target set. Interaction pairs of miRNAs were identified through filtering based on interaction with a measurement of 0 or without common features (e.g., environmental chemical compounds, small molecular drugs). Thereafter, a miRNA crosstalk network was constructed.

## 3. Results

### 3.1. The Flowchart of the Present Study

**Figure 1:**
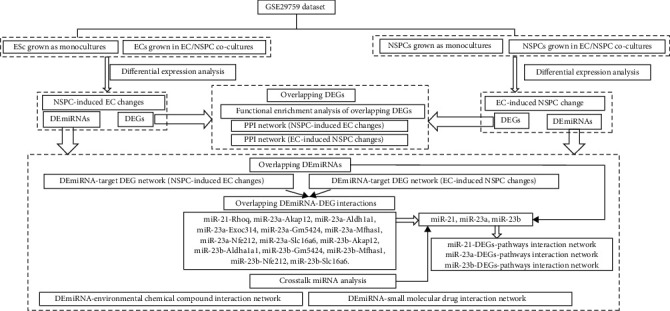
The flowchart of the present study.

The study design of the present research is shown in Figure 1. As shown in Figure 1, the differential expression analysis was performed to investigate the DEGs and DEmiRNAs involved in the NSPC-induced EC alteration and EC-induced NSPC alteration. As regards DEGs, the DEGs overlapped between DEGs involved in NSPC-induced EC alteration and EC-induced NSPC alteration were identified, and then the functional enrichment analysis was performed to investigate the biological processes and signaling pathways in which these overlapping DEGs were involved. Based on the DEGs, respectively, involved in the NSPC-induced EC alteration and EC-induced NSPC alteration, the corresponding PPI network was constructed. As regards DEmiRNAs, the DEmiRNAs overlapped between DEmiRNAs expressed in the NSPC-induced EC alteration and EC-induced NSPC alteration were identified to be mmu-miR-23a, mmu-miR-23b, and mmu-miR-210. Afterward, the DEmiRNA-DEG interaction networks were constructed, respectively, for the NSPC-induced EC alteration and EC-induced NSPC alteration, from which the overlapping DEmiRNA-DEG interaction pairs were found. The crosstalk miRNA analysis was performed and validated the crosstalk role of three DEmiRNAs (mmu-miR-23a, mmu-miR-23b, and mmu-miR-210) in the interaction between ECs and NSPCs. The corresponding miRNA-DEG-pathway network, respectively, for these three DEmiRNAs was constructed. In addition, the chemical compounds and small molecular drugs which regulate the expression patterns of DEmiRNAs were also identified by constructing the DEmiRNA-environmental chemical compound interaction network and DEmiRNA-small molecular drug interaction network.

### 3.2. Identification of DEGs and DEmiRNAs

**Figure 2:**
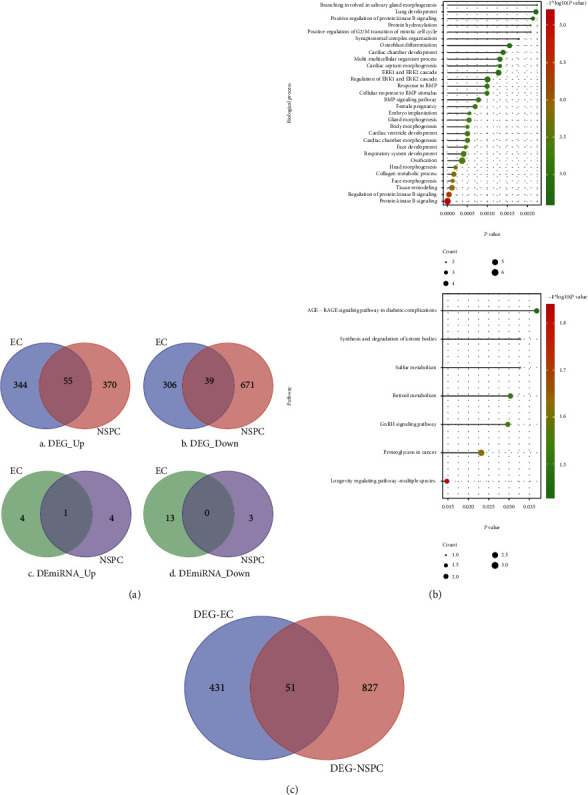
The DEGs identified to be involved in the EC-induced NSPC alterations and NSPC-induced EC alterations and the functions in which these DEGs were involved. (a) Venn diagram showing the overlapped DEGs and DEmiRNAs that hold the same expression patterns (either up- or downregulated) in EC-induced NSPC alterations and NSPC-induced EC alterations. (b) The top 30 BPs (A) and top signaling pathways (B) in which overlapped DEGs were enriched. The overlapped DEGs are shared between the NSPC-induced EC alterations and the EC-induced NSPC alterations. (c) The Venn diagram shows the DEGs overlapped between the DEGs involved in the EC-PPI network (PPI network involved in the NSPC-induced EC genetic alteration) and the NSPC-PPI network (PPI network involved in the EC-induced NSPC genetic alteration).

Table 1 and Figure 2(a) show that 94 DEGs consisting of 55 upregulated DEGs and 39 downregulated DEGs were overlapping. Tables 2 and 3 list the differential expression values (e.g., logFC, AveExpr, *t* value, *P* value, adj. *P* value, *B* value, and regulation pattern (up/down)) of the top 30 DEGs which were, respectively, differentially expressed in the NSPC-induced EC alteration and EC-induced NSPC alteration. Three DEmiRNAs found to be overlapped between the EC-induced NSPC alteration and NSPC-induced EC alteration categories (i.e., mmu-miR-23a, mmu-miR-23b, and mmu-miR-210) were selected for further investigation. The expression pattern of mmu-miR-210 was similar in both EC-induced NSPC alterations and NSPC-induced EC alterations. However, the expression patterns of mmu-miR-23a and mmu-miR-23b differed in both situations.

### 3.3. Functions of Overlapping DEGs

Figure 2(b) depicts the biological processes and pathways in which overlapping DEGs were enriched. As seen in Figure 2(b) A, the overlapping DEGs were found to be involved in several biological processes, for instance, positive regulation of protein kinase B (PKB, also known as Akt), ERK1/2 cascade, response to BMP, collagen metabolic process, and tissue remodeling. The overlapping DEGs were found to be involved in several signaling pathways including synthesis and degradation of ketone bodies, sulfur metabolism, retinol metabolism, and GnRH signaling (Figure 2(b) B).

### 3.4. EC-PPI Network and NSPC-PPI Network

**Figure 3:**
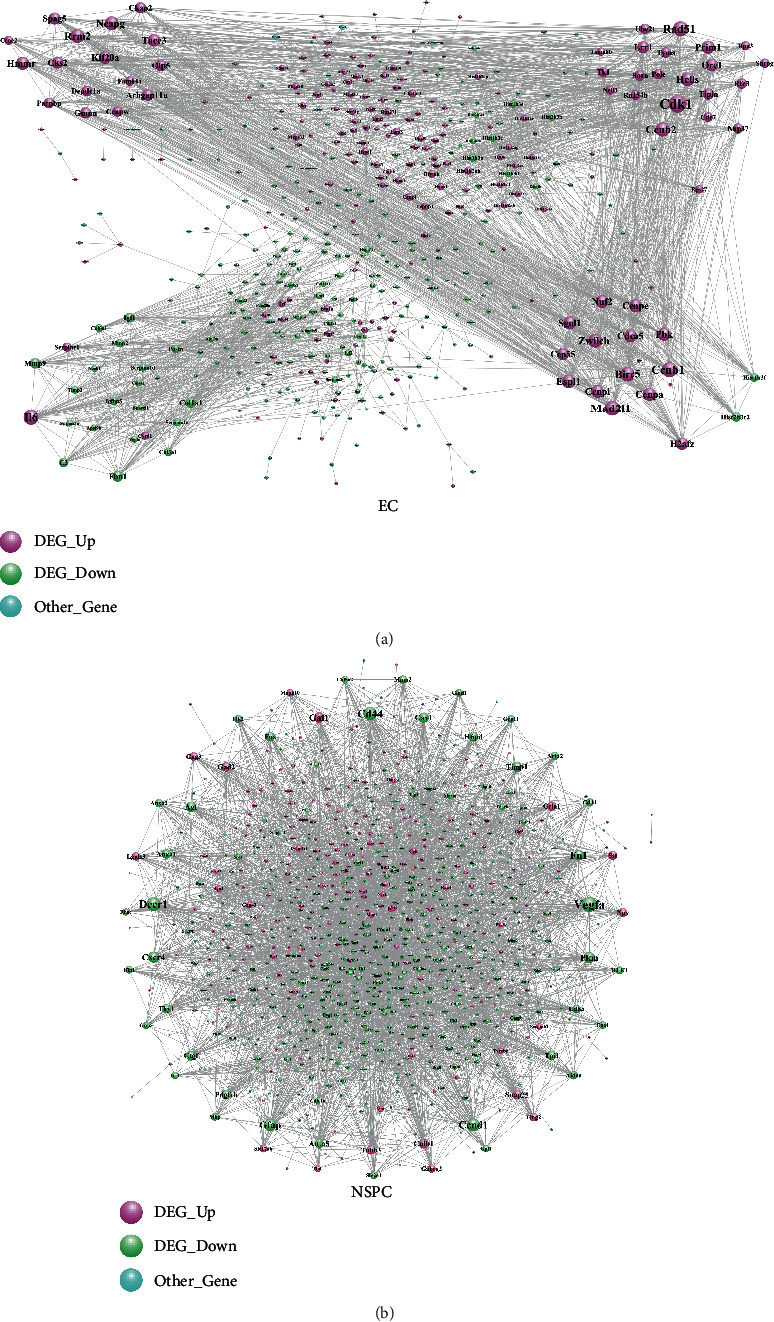
The PPI network of DEGs involved in NSPC-induced EC alteration (a) and DEGs involved in EC-induced NSPC alteration (b). Other genes are non-DEGs.

The PPI network consisting of DEGs involved in NSPC-induced EC alteration (EC-PPI network) was constructed and included 482 nodes with 2,679 edges (Figure 3(a)), while the PPI network consisting of DEGs involved in EC-induced NSPC alteration (NSPC-PPI network) showed 878 nodes and 3,795 edges (Figure 3(b)). The characteristics of the top 30 nodes of EC-PPI as well as NSPC-PPI network are listed in descending order of degree (Tables 4 and 5).

By construction of a Venn diagram, 51 DEGs were overlapping between the EC-PPI network and the NSPC-PPI network (Figure 2(c)). The topological features of the overlapping DEGs in the EC-PPI network and the NSPC-PPI network are listed in Tables 6 and 7, respectively.

### 3.5. The Transcription Factor-Target DEG Interaction Network

**Figure 4:**
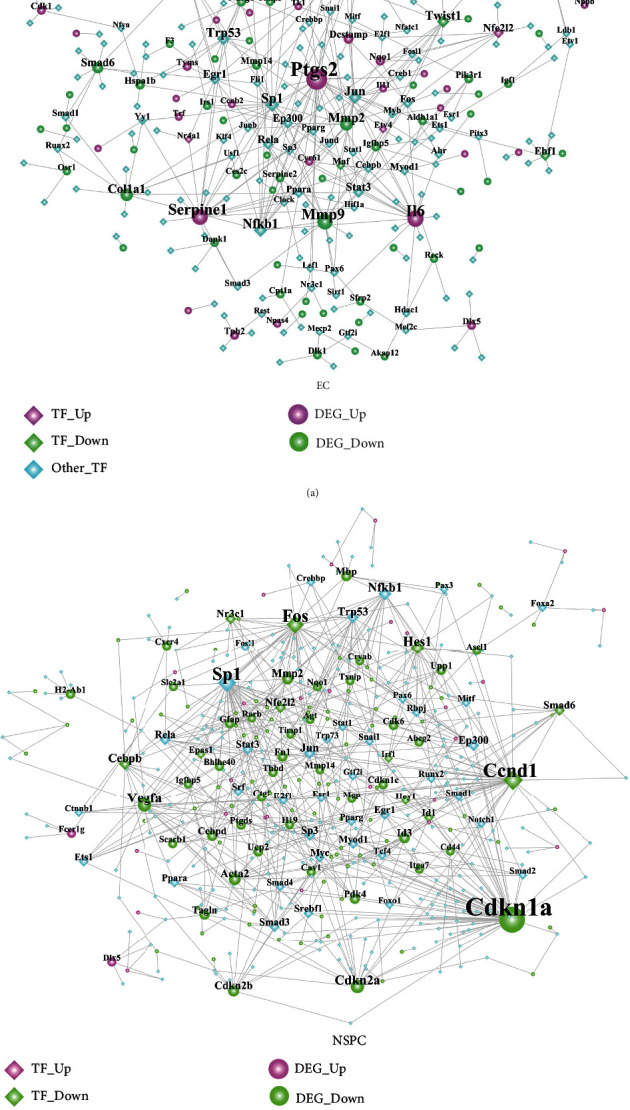
The transcription factor-target DEG interaction network. (a) The TF-target DEG network involved in the NSPC-induced EC alteration. (b) The TF-target DEG network involved in the EC-induced NSPC alteration.

The TF-target DEG network was constructed, respectively, for the DEGs involved in the NSPC-induced EC alteration (Figure 4(a)) and EC-induced NPSC alteration (Figure 4(b)). Figure 4(a) consists of 285 nodes and 363 edges, while Figure 4(b) consists of 451 nodes and 668 edges. As shown in Figure 4(a) and Table 8, several TFs (e.g., Sp1, Nfkb1, Trp53, Jun, Twist1, and Stat3) were identified to be hub transcription factors targeting the greatest number of DEGs involved in the NSPC-induced EC alteration. As shown in Figure 4(b) and Table 9, several TFs (e.g., Ccnd1, Sp1, Fos, Hes1, Nfkb1, and Cebpb) were identified to be hub transcription factors targeting the greatest number of DEGs involved in the EC-induced NPSC alteration.

### 3.6. The DEmiRNAs-Target DEGs and Their Functions

**Figure 5:**
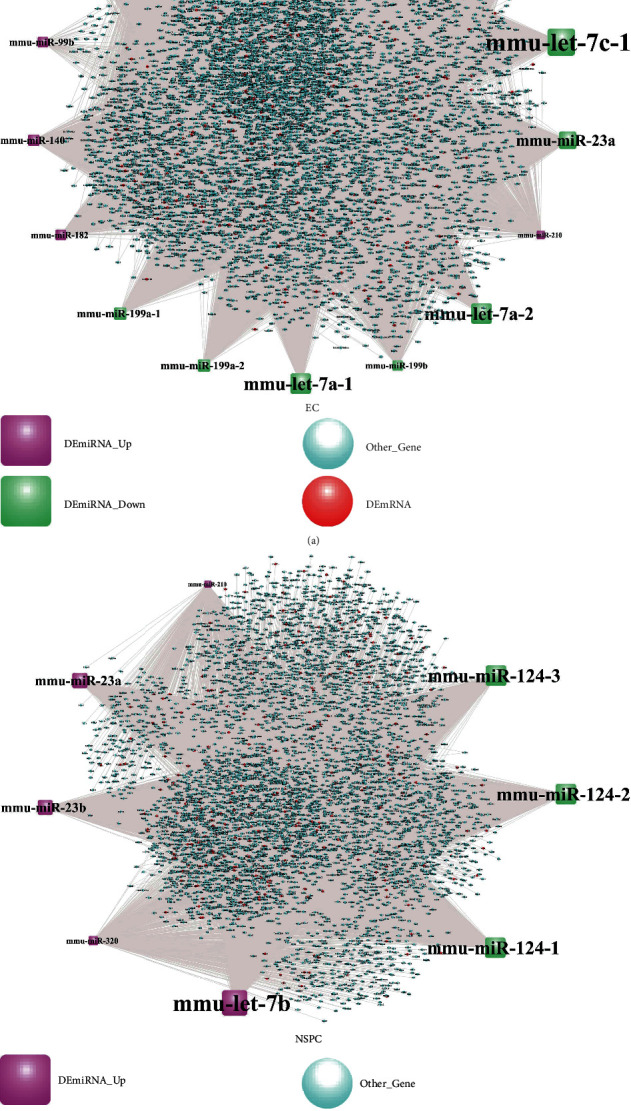
The DEmiRNA-target network involved in the NSPC-induced EC alteration (a) and EC-induced NSPC alteration (b).

The number of experimentally validated, computationally predicted, and total miRNA-DEtargets for ECs and NSPCs is listed in Table 10. As evident here, 14 interaction pairs of miRNA-DEtargets were determined. The DEmiRNA-target network involved in NSPC-induced EC alteration was constructed and included 5,916 nodes with 22,664 edges (Figure 5(a)), while the DEmiRNA-target network involved in NSPC-induced EC alteration showed 4,962 nodes and 11,675 edges (Figure 5(b)). The characteristics of the top 30 nodes of these two networks are listed in descending order of degree (Tables 11 and 12).

**Figure 6:**
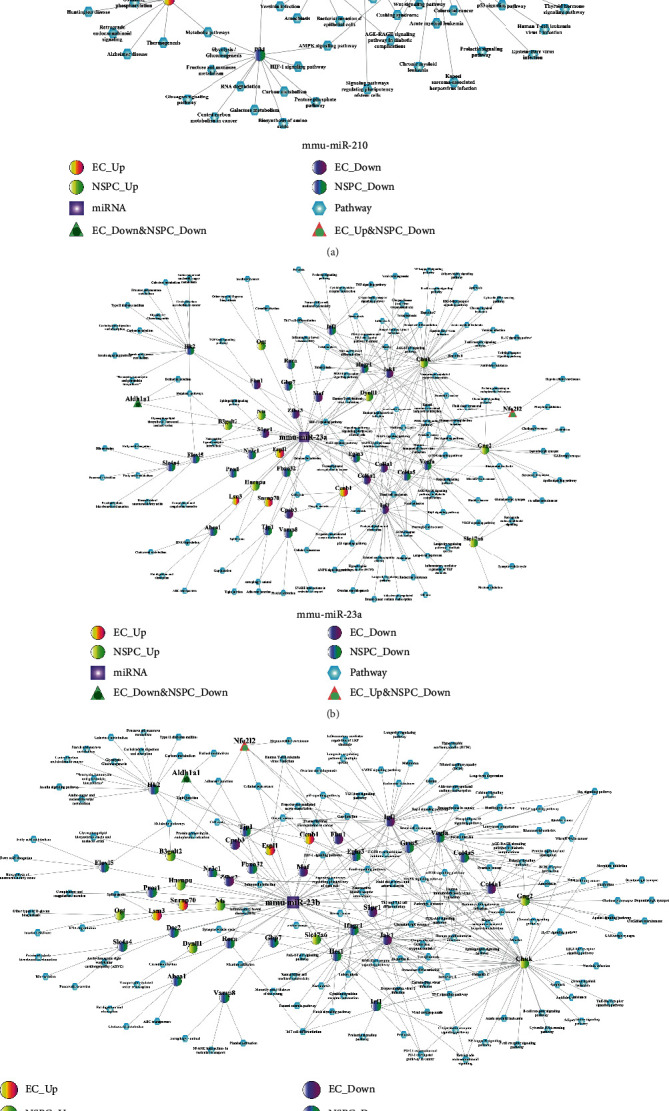
The miRNA-DEG-pathway interaction network. (a) mmu-miR-210, (b) mmu-miR-23a, and (c) mmu-miR-23b.

The miR-DEG-pathway network for three selected miRNAs (miR-210 (Figure 6(a)), miR-23a (Figure 6(b)), and miR-23b (Figure 6(c))) is depicted in Figure 6. As shown in Figure 6(a), miR-210 targets the Rhoq gene which was found to be downregulated in both NSPC-induced EC alteration and EC-induced NSPC alteration. The Rhoq gene was shown to be involved in the insulin signaling pathway. As shown in Figure 6(b), miR-23a targets the Aldh1a1 gene which was found to be downregulated in both NSPC-induced EC alteration and EC-induced NSPC alteration. The Aldh1a1 gene was shown to be involved in the retinol metabolism and metabolic pathway. It could be observed from Figure 6(c) that the target genes of miR-23b were almost the same as those of miR-23a. miR-23b can target the Igf1 gene which was found to be downregulated in NSPC-induced EC alteration. The Igf1 gene was involved in many signaling pathways including AMPK, Rap1, HIF-1, p53, and FoxO signaling.

### 3.7. Identifying Significant Small Molecular Drugs

**Figure 7:**
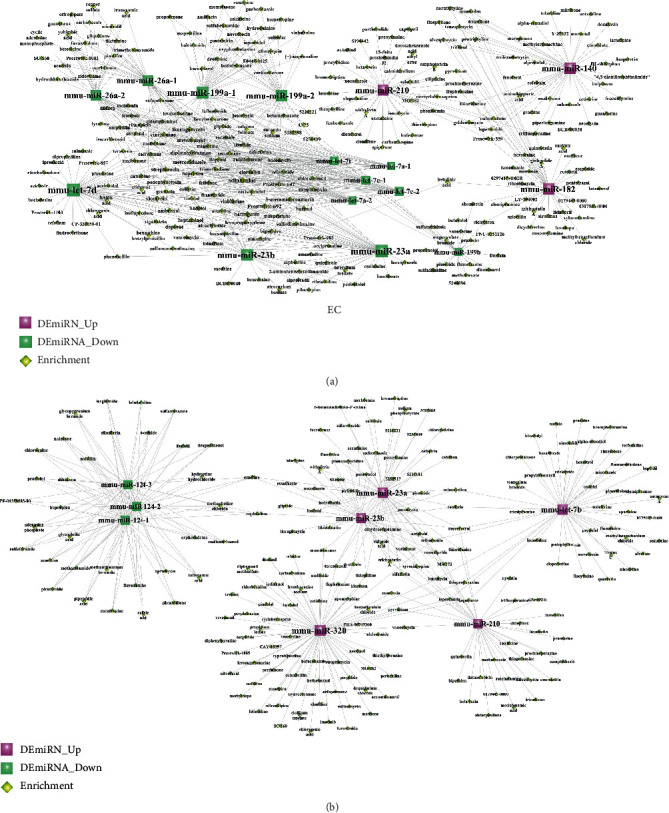
The DEmiRNA-small molecular drug network involved in the NSPC-induced EC alterations (a) and EC-induced NSPC alterations (b). Red squares represent upregulated miRNAs, green squares indicate downregulated miRNAs, and yellow diamonds indicate miRNA-related small molecular drugs.

Figure 7 shows the DEmiRNA-small molecular drug network involved in the NSPC-induced EC alterations (Figure 7(a)) and EC-induced NSPC alterations (Figure 7(b)). Upon comparing the small molecules relevant to NSPC-induced EC alteration and EC-induced NSPC alteration, a total of 16 small drug molecules were found to be overlapped and significantly enriched (Table 13). Table 13 shows that 6 small molecular drugs (e.g., deptropine, fluphenazine, lycorine, quinostatin, resveratrol, and thiamazole) had similar regulatory roles in the process of EC-induced NSPC alteration and NSPC-induced EC alteration, indicating simultaneous upregulation or downregulation.

### 3.8. The miRNA-Environmental Chemical Compound Network

**Figure 8:**
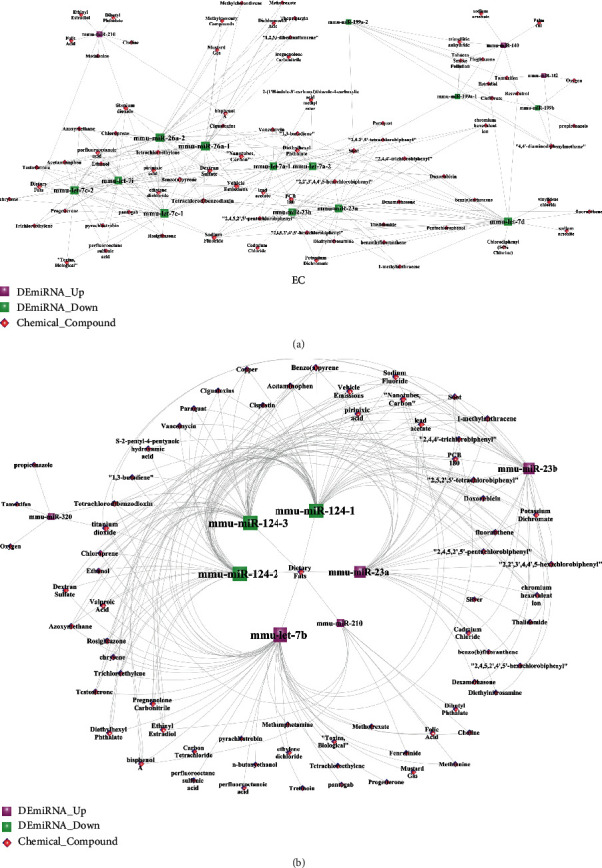
The DEmiRNA-chemical compound network involved in the NSPC-induced EC alterations (a) and EC-induced NSPC alterations (b). Red squares represent upregulated miRNAs, green squares indicate downregulated miRNAs, and pink diamonds indicate miRNA-related chemical compounds.

There were 60 environmental chemical compounds highly correlated with the DEmiRNAs expressed during NSPC-induced EC alteration and EC-induced NSPC alteration (Figure 8). As shown from Figure 8, miR-210 was significantly related to many compounds such as bisphenol A, titanium dioxide, choline, dietary fats, folic acid, and methionine. miR-23b was found to be significantly related to many compounds such as dexamethasone, potassium dichromate, sodium fluoride, thalidomide, cadmium chloride, and soot.

### 3.9. Crosstalk miRNAs Linking the EC-Induced NSPC Alteration Process and NSPC-Induced EC Alteration Process

**Figure 9:**
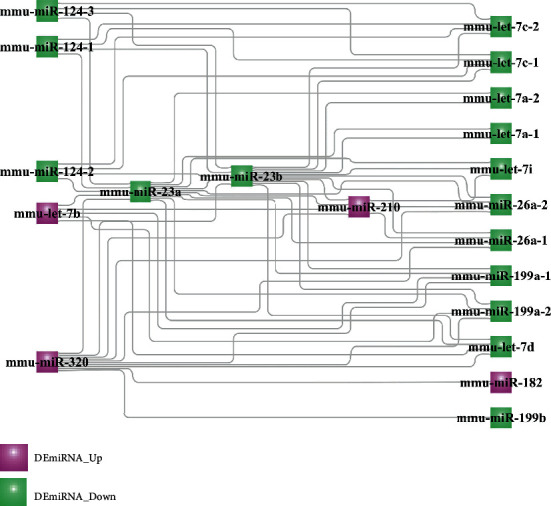
The crosstalk DEmiRNA interaction network involved in the link between the EC-induced NSPC alteration process and the NSPC-induced EC alteration process.

A total of 54 interaction pairs of miRNAs were determined and are listed in Table 14. As shown in Figure 9, three miRNAs (miR-23a, miR-23b, and miR-210) were determined to perform critical bridging roles in the link between the EC-induced NSPC alteration process and the NSPC-induced EC alteration process.

## 4. Discussion

This study identified three miRNAs (i.e., miR-210, miR-230a, and miR-23b) to be crosstalks involved in the interaction between ECs and NSPCs. The roles of these miRNAs in regulating the interactions between ECs and NSPCs will be discussed in the following sections by using the previous related evidence.

miR-210 is identified to be involved in the interaction between ECs and NSPCs by targeting many genes (e.g., Rhoq, Fn1, and Pard3) and pathways (e.g., insulin, PI3K/Akt, and chemokine signaling pathway) (Figure 3(a)), and its regulating roles will be discussed in this and next paragraph. Rhoq (Ras Homolog (Rho) Family Member Q) was identified to be downregulated during the process of EC-induced NSPC alteration and NSPC-induced EC alteration; however, Rhoq has not been investigated to regulate NSPCs and ECs. As another member of the Rho family, RhoA was found to influence the proliferation and fate of NSPCs by mediating mechanotransduction and cellular stiffness [29, 30], as well as impact the migration and angiogenesis of ECs by mediating the cytoskeletal changes [31]. The insulin signaling pathway targeted by RhoA could promote angiogenic processes via mediating the migration and proliferation of ECs as well as in vitro tubular structure formation [32]. The insulin-mediated pathway could also regulate the self-renewal, neurogenesis, cognition, and sensory function, as well as homeostasis of NSPCs [33].

During the interactions between ECs and NSPCs, the genes dysregulated by a certain type of cell might influence the biological behavior and processes of another type of cell. For instance, the addition of ECs into NSPCs induced the downregulation of the gene Fn1 (fibronectin-1) in NSPCs. While fibronectin was shown to regulate the survival and migration of primary NSPCs [34], its dysregulation in NSPCs can modulate the functions of ECs by influencing survival and angiogenesis in both the integrin-dependent and integrin-independent manners [35]. The PI3K/Akt pathway targeted by Fn1 has been established as a classic pathway in regulating both angiogenesis and neurogenesis. This pathway plays a mediating role in neural regeneration by controlling the survival, proliferation, differentiation, and migration of NSPCs [36]. The link between this pathway and angiogenesis has been highlighted since it can modulate the expression of many angiogenic factors such as vascular endothelial growth factor (VEGF), nitric oxide, and angiopoietins [37]. As another example, the addition of NSPCs to ECs induced a decreased expression of the gene Pard3 (Par-3 Family Cell Polarity Regulator) in ECs. Pard3 can not only determine the cell polarity of ECs [38] but also modulate the sprouting behavior of ECs during the process of angiogenesis [39]. Conversely, the dysregulation of cell polarity regulators can influence the behavior and functions of NSPCs in terms of architecture and shape, interkinetic nuclear migration, proliferation and differentiation potential, and asymmetric cell division [40]. The chemokine signaling pathway targeted by Pard3 was shown to promote neovascularization by stimulating the migration and proliferation of ECs, as well as recruiting endothelial progenitor cells [41]. Regarding the role of chemokine in regulating NSPCs, chemokines were shown to promote the quiescence and survival of NSPCs [42], as well as regulate the migration of NSPCs toward the sites of neuroinflammation [43]. Overall, based on this existing evidence, it can be assumed that miR-210 plays a critical role in the interaction between ECs and NSPCs by targeting genes and pathways associated with both neurogenesis and angiogenesis.

The mechanisms of two miRNAs (miR-23a and miR-23b) involved in the interaction between ECs and NSPCs are almost similar, and the regulatory roles of miR-23a will be therefore an example to discuss in this and next paragraph. As shown in Figure 3(b), miR-23a is involved in the interaction between ECs and NSPCs by targeting many genes (e.g., Aldh1a1, Fbn1, and Chuk) and pathways (e.g., retinol metabolism, TGF-beta, and NF-*κ*B pathway). For example, the Aldh1a1 (Aldehyde Dehydrogenase 1 Family Member A1) gene targeted by miR-23a was found to be simultaneously downregulated during the process of EC-induced NSPC alteration and NSPC-induced EC alteration. High activity of aldehyde dehydrogenase (ALDH) in tumor ECs and its function in promoting angiogenesis in tumor progression have been reported [44]; however, so far, there is no evidence supporting the angiogenic role of ALDH in stem cell-like ECs. A recent review [45] reported that aldehyde dehydrogenase (ALDH) not only represents an intracellular and metabolic marker of stem cells including NSPCs and endothelial progenitor cells (EPCs) but also acts as a functional regulator of stem cells mainly by mediating the metabolic pathway of retinoic acid (RA). The retinol metabolism pathway targeted by Aldh1a1 was shown to be involved at the early stage of neurogenesis and antiangiogenic response. In terms of neurogenesis, RA has been commonly applied for differentiating NSPCs due to its function in promoting the acquisition of a neuronal fate [46]. In terms of antiangiogenic response, RA has been shown to selectively block vascular permeability factor/vascular endothelial growth factor (VPF/VEGF), thereby further inhibiting these angiogenic factors that induced microvascular permeability [47].

The interaction of ECs and NSPCs supports the angiogenesis of ECs by influencing the expression of angiogenesis-related genes in ECs; likewise, this interaction also supports the neurogenesis of NSPCs by impacting the neurogenesis-associated genes in NSPCs. For instance, the addition of NSPCs into ECs resulted in the downregulation of Fbn1 (Fibrillin 1) in ECs. The encoded protein of gene Fbn1 is a large extracellular matrix (ECM) glycoprotein, which assembles to form 10-12 nm microfibrils in ECM. ECM has been demonstrated as a hard player in regulating angiogenesis via interacting/restoring a variety of growth factors (e.g., fibroblast growth factor (FGF), platelet-derived growth factor (PDGF), hepatocyte growth factor (HGF), and VEGF) [48]. In addition to its involvement in angiogenesis, ECM was shown to regulate the proliferation and differentiation of NSPCs by inducing the local alterations in the substrate stiffness [30]. The TGF-beta signaling pathway targeted by Fbn1 has been regarded as an indispensable player in facilitating interactions between ECs and NSPCs based on its function in vasculogenesis and angiogenesis by upregulating the expression of angiogenic factors [49], as well as its function in neurogenesis by modulating the temporal specification and progenitor potency of NSPCs [50]. As another example, the addition of ECs into NSPCs was noted to cause the upregulation of the gene Chuk (Component of Inhibitor of Nuclear Factor Kappa B Kinase) in NSPCs. The encoded protein of Chuk is a component of a cytokine-activated protein complex that acts as an inhibitor of the transcription factor NF-*κ*B complex [51]. The Chuk gene overexpressed in NSPCs might be a crosstalk gene in promoting interactions between NSPCs and ECs, based on the function of the Chuk-targeted NF-*κ*B pathway in regulating neurogenesis [52] and angiogenesis [53]. The activation of the NF-*κ*B pathway is known to be involved in angiogenesis by increasing the proliferating potential of vascular ECs and increasing the expression levels of vascular growth factors [53]. In addition, the regulatory function of the NF-*κ*B pathway in neurogenesis is reflected in its role in initiating the early differentiation of NSPCs [52]. In summary, previous evidence supports the notion that miR-23a plays a role in promoting the interactions between ECs and NSPCs by targeting genes and pathways relevant to angiogenesis and neurogenesis.

The research value of the current research should be highlighted by the comparison between the previously published paper based on the same dataset GSE29759 [10] and the results obtained by the current research. In the previously published paper [10], the authors performed differential expression analysis and have listed the up- and downregulated DEmiRNAs which were differentially expressed in NSPC-induced EC alteration and EC-induced NSPC alteration, respectively. However, further deep investigation about the crosstalk miRNAs linking the interaction between NSPCs and ECs is lacking. In order to remedy this research gap, the present research made full use of this valuable dataset with reasonable study design by performing a series of comprehensive bioinformatics analysis and finally obtained the main results showing the crosstalk role of three miRNAs (miR-210, miR-230a, and miR-23b) in the reciprocal interaction between ECs and NSPCs. Apart from this main finding, the present research also identified that some other genetic biomarkers (i.e., six genes (i.e., MMP14, TIMP3, LOXL1, CCK, SMAD6, and HSPA2) and three pathways (i.e., Akt, ERK1/2, and BMPs)) play critical roles in the interaction of ECs and NSPCs. And also, some target drugs (six small molecular drugs (i.e., deptropine, fluphenazine, lycorine, quinostatin, resveratrol, and thiamazole) and seven environmental chemical compounds (i.e., folic acid, dexamethasone, choline, doxorubicin, thalidomide, bisphenol A, and titanium dioxide)) were identified to influence angiogenesis and neurogenesis by regulating the expression of DEmiRNAs. These results obtained by the current research not only fully explained the underlying information of the dataset GSE29759 but also facilitated neurology researchers to have a better understanding of the genetic and epigenetic mechanisms involved in the interaction between ECs and NSPCs.

It is important to acknowledge the limitation of this research. The first limitation is lacking experimental validation using quantitative real-time polymerase chain reaction (qRT-PCR) to verify the dysregulation of biomarkers identified during the reciprocal alterations between ECs and NPSCs; however, investigating the expression patterns and detailed functions of these biomarkers (especially 3 miRNAs (miR-210, miR-230a, and miR-23b)) as the crosstalks between ECs and NPSCs could be a separate research study and within the research plan of our team. The second limitation is only one dataset (GSE29759) related to this research topic was included, which means the sample size that was analyzed was comparatively limited. This indicates that the future RNA-sequencing study could follow the same study design; if so, more datasets could be combined and analyzed together, which could increase the prediction accuracy of the results identified by bioinformatics analyses. The third limitation is the dataset (GSE29759) only examined the messenger RNA and miRNA expression profiles. Since noncoding RNAs contain miRNAs, long noncoding RNAs (lncRNAs), circular RNAs (circRNAs), and Piwi-interacting RNA (piRNA), the future research with the same study design could further examine the other noncoding RNA expression profile alterations that occurred during the reciprocal changes in the interactions between ECs and NPSCs. If more datasets with the design of examining the other noncoding RNA expression profiles were included in this analysis, then constructing a circRNA/lncRNA-miRNA-mRNA competitive endogenous RNA (ceRNA) interaction network will be more meaningful for investigating the genetic and epigenetic crosstalks involved in the reciprocal interactions between ECs and NPSCs.

Although limitations exist in this research, the present study also provides implications for future research. First, the genetic and epigenetic linkage mechanisms as well as drugs that are involved in the interactions between ECs and NSPCs could translate to novel gene/drug delivery strategies for constructing 3D neurovascular tissue. With the rapid advancements in genome-editing techniques, it may be possible to perform the gene modifications on stem cells (i.e., endothelial stem-like cells and NSPCs) specific to the crosstalk genes and miRNAs identified in this study. The small molecular drugs and chemical compounds are relevant to nanoparticle research for controlled drug delivery to exert sustained effects on the neurogenic and angiogenic processes of ECs and NSPCs. Another noteworthy issue that should be investigated is the dose and concentration of drugs and chemical compounds, especially considering their toxicity and degradation rate. In addition, several of the identified entities are candidates for future research for the purpose of experimental validation, as these have not been investigated in the context of interactions between ECs and NSPCs.

In addition, the present research has potential clinical transfer values for ischemic stroke therapy. Since the current clinical treatments (e.g., intravenous administration of tissue-type plasminogen activator, early motor training, and physical therapy) could not result in complete functional recovery in ischemic stroke patients [54], construction of a multicellular EC-NPSC-based 3D neurovascular unit model might be an alternative tissue engineering approach for regeneration of stroke-affected neuronal tissue. However, there are still no clinical trials using the biomaterial scaffolds carried with ECs and NPSCs in stroke therapy. Although tissue engineering is currently fraught with many challenges [55, 56], the identification of genetic and epigenetic mechanisms and drugs in facilitating the interactions between ECs and NSPCs might be potentially used for the genetic/epigenetic modification of ECs/NSPCs and drug delivery of biomaterial scaffolds and thereby enhance the effects of angiogenesis and neurogenesis. By performing these modifications, the success rate of constructing a 3D neurovascular unit model might be significantly increased. Therefore, the findings of the present research may be regarded as a preliminary step providing a theoretical basis to further research toward a promising stem cell-based stroke therapy.

## 5. Conclusions

This bioinformatics study identified 3 miRNAs (miR-210, miR-230a, and miR-23b) as crosstalk entities involved in the interaction between ECs and NSPCs. These 3 miRNAs may be relevant to the arena of stem cell modification to further promote angiogenesis and neurogenesis within the field of stem cell tissue engineering.

## Figures and Tables

**Table 1:** The number of up- and downregulated DEGs and DEmiRNAs that are, respectively, involved in NSPC-induced EC alterations and EC-induced NSPC alterations.

Coculture-induced genetic and epigenetic alteration in one type of cells	Number of DEmRNAs (DEGs)			Number of DEmiRNAs		
	Upregulated DEGs	Downregulated DEGs	Total	Upregulated DEmiRNAs	Downregulated DEmiRNAs	Total
NSPC-induced alterations in ECs	399	345	744	5	13	18
EC-induced alterations in NSPCs	425	710	1135	5	3	8

**Table 2:** The top 30 DEGs involved in the NSPC-induced alterations in ECs, ranked by the ascending order of *P* value.

Gene	logFC	AveExpr	*t* value	*P* value	Adj. *P* value	*B* value	Regulation pattern
Matn2	-0.95204	0.491256	-10.0494	3.86*E* − 06	0.025782	4.509474	Down
Fmod	-1.02587	0.511182	-10.011	3.98*E* − 06	0.025782	4.485352	Down
LOC102641248///Maf	-1.1636	0.572078	-9.81371	4.69*E* − 06	0.025782	4.359141	Down
Csf2	0.946405	-0.49723	9.80391	4.73*E* − 06	0.025782	4.352778	Up
Cyr61	1.916043	-0.90785	9.163675	8.19*E* − 06	0.035735	3.917772	Up
Rad54b	0.884623	-0.50864	8.718932	1.22*E* − 05	0.038883	3.591553	Up
Hmgb3	1.027877	-0.53931	8.697135	1.25*E* − 05	0.038883	3.575027	Up
Srpx2	-1.03053	0.491458	-8.532	1.45*E* − 05	0.039663	3.448146	Down
Ces1g	-1.12288	0.585271	-8.39761	1.65*E* − 05	0.040014	3.342658	Down
Glis3	-0.82208	0.370708	-8.27324	1.86*E* − 05	0.040548	3.243204	Down
Negr1	-0.85748	0.4562	-8.03571	2.34*E* − 05	0.041325	3.048246	Down
C1ra	-0.80236	0.398421	-7.9815	2.47*E* − 05	0.041325	3.002814	Down
Smarca1	-0.90427	0.471865	-7.90938	2.65*E* − 05	0.041325	2.941813	Down
Gmnn	0.868301	-0.43587	7.834033	2.86*E* − 05	0.041325	2.877409	Up
Tex30	1.021454	-0.5467	7.64017	3.48*E* − 05	0.041325	2.708438	Up
Steap4	-0.90097	0.356113	-7.58391	3.68*E* − 05	0.041325	2.658512	Down
Rbms3	-0.79877	0.399764	-7.5764	3.71*E* − 05	0.041325	2.651814	Down
Cdo1	-0.87123	0.459852	-7.51396	3.96*E* − 05	0.041325	2.595861	Down
Col3a1	-0.72967	0.358484	-7.49893	4.02*E* − 05	0.041325	2.582317	Down
3632451O06Rik	-0.99083	0.395715	-7.37964	4.55*E* − 05	0.041325	2.47378	Down
C3	-0.8261	0.446644	-7.36875	4.61*E* − 05	0.041325	2.463774	Down
Thsd7a	-1.00741	0.566198	-7.34217	4.74*E* − 05	0.041325	2.439301	Down
Mad2l1	0.769878	-0.43152	7.272629	5.10*E* − 05	0.041325	2.374807	Up
Ccne2	0.891927	-0.50969	7.268195	5.12*E* − 05	0.041325	2.370674	Up
Adamts2	-0.9331	0.460263	-7.23757	5.29*E* − 05	0.041325	2.342049	Down
AI506816	0.79492	-0.41645	7.222375	5.38*E* − 05	0.041325	2.327797	Up
Timm8a1	0.742468	-0.41919	7.211119	5.44*E* − 05	0.041325	2.317221	Up
Ccnb2	0.6736	-0.32421	7.16572	5.71*E* − 05	0.041325	2.274388	Up
Tyms///Tyms-ps	0.676379	-0.36727	7.152192	5.80*E* − 05	0.041325	2.26157	Up
5730414N17Rik	-0.79662	0.418908	-7.14794	5.82*E* − 05	0.041325	2.257534	Down

**Table 3:** The top 30 DEGs involved in the EC-involved alterations in NSPCs, ranked by the ascending order of *P* value.

Gene	logFC	AveExpr	*t* value	*P* value	Adj. *P* value	*B* value	Regulation pattern
Pcolce2	-3.29485	1.6351	-45.4871	2.08*E* − 11	4.55*E* − 07	13.2377	Down
Dbp	-2.00859	1.062409	-26.2015	2.14*E* − 09	2.34*E* − 05	11.11723	Down
AU020206	-1.63296	0.821066	-21.5827	1.08*E* − 08	7.32*E* − 05	10.05187	Down
F3	-1.6611	0.850706	-20.9105	1.41*E* − 08	7.32*E* − 05	9.863187	Down
Tpi1	-1.68121	0.830618	-20.4734	1.68*E* − 08	7.32*E* − 05	9.73502	Down
Gm2115	-1.53464	0.719453	-19.4163	2.60*E* − 08	9.46*E* − 05	9.405935	Down
Ldha	-1.78095	0.900045	-18.2662	4.32*E* − 08	0.000134	9.014231	Down
Sorl1	-1.45704	0.706881	-17.7044	5.59*E* − 08	0.000135	8.808873	Down
Dct	-1.30765	0.669351	-17.6998	5.60*E* − 08	0.000135	8.807132	Down
Klf9	-1.86306	0.937896	-17.4915	6.17*E* − 08	0.000135	8.728473	Down
Pgk1	-1.18385	0.609345	-16.5411	9.78*E* − 08	0.000194	8.351213	Down
Gypc	-1.22407	0.595138	-16.039	1.26*E* − 07	0.000207	8.139031	Down
Aldoa	-1.26041	0.65786	-15.9803	1.30*E* − 07	0.000207	8.113603	Down
Plekhf1	-1.317	0.690027	-15.8197	1.41*E* − 07	0.000207	8.043409	Down
4-Sep	-1.14394	0.579925	-15.6971	1.50*E* − 07	0.000207	7.989137	Down
Aldh1a1	-2.80632	1.506176	-15.6785	1.52*E* − 07	0.000207	7.980857	Down
Plin3	-1.2668	0.589403	-15.1096	2.06*E* − 07	0.000264	7.720663	Down
Parp9	-1.23876	0.648245	-14.7499	2.50*E* − 07	0.000303	7.549197	Down
Cldn12	-1.28813	0.622674	-14.0937	3.63*E* − 07	0.000408	7.221524	Down
Gpi1	-1.00716	0.517518	-14.0417	3.74*E* − 07	0.000408	7.194653	Down
Htra1	-1.19764	0.56397	-13.8878	4.09*E* − 07	0.000409	7.114509	Down
Srd5a1	-1.16578	0.561656	-13.8718	4.13*E* − 07	0.000409	7.106129	Down
Dlx5	1.127851	-0.58715	13.43145	5.37*E* − 07	0.000478	6.87001	Up
ND5	1.058484	-0.52334	13.36773	5.58*E* − 07	0.000478	6.835023	Up
Rbp1	-1.12775	0.585747	-13.3416	5.67*E* − 07	0.000478	6.820608	Down
Atp1b2	-1.03892	0.500454	-13.3344	5.69*E* − 07	0.000478	6.816639	Down
Clybl	-1.27083	0.618039	-13.1367	6.42*E* − 07	0.000519	6.706417	Down
Sox8	-1.02853	0.485142	-12.9808	7.08*E* − 07	0.000551	6.617969	Down
Srprb///Trf	-1.18478	0.624131	-12.8262	7.80*E* − 07	0.000586	6.52904	Down
Hspb6	-0.93188	0.490732	-12.7078	8.40*E* − 07	0.000594	6.459985	Down

**Table 4:** The topological characteristics of the top 30 nodes in the PPI network of NSPC-induced EC genetic alterations. NA means not overlapped between the EC-PPI and NSPC-PPI networks.

DEGs	Overlap	Regulate	Degree	Average shortest path length	Betweenness centrality	Closeness centrality	Topological coefficient
Cdk1	NA	Up	72	2.737069	0.056051	0.365354	0.179894
Mad2l1	NA	Up	63	2.844828	0.019572	0.351515	0.211029
Ccnb1	NA	Up	60	2.706897	0.084386	0.369427	0.195539
Il6	NA	Up	58	2.872845	0.111663	0.348087	0.08938
Rad51	NA	Up	58	2.855603	0.04335	0.350189	0.232014
Birc5	NA	Up	56	2.732759	0.058321	0.365931	0.213803
Ccnb2	NA	Up	54	2.922414	0.006045	0.342183	0.255649
Ncapg	NA	Up	52	3.032328	0.008236	0.32978	0.282051
Rrm2	NA	Up	52	2.931034	0.018658	0.341176	0.262327
Zwilch	NA	Up	52	2.974138	0.008695	0.336232	0.26455
Espl1	NA	Up	51	2.961207	0.010195	0.3377	0.265609
Hells	NA	Up	51	2.967672	0.006265	0.336964	0.268318
Cdca5	NA	Up	49	2.971983	0.002445	0.336476	0.279941
Cenpa	NA	Up	49	2.997845	0.016442	0.333573	0.260875
Cenpe	NA	Up	49	2.961207	0.018044	0.3377	0.257355
Hmmr	NA	Up	49	3.040948	0.009178	0.328845	0.288843
Pbk	NA	Up	49	3.006466	0.002026	0.332616	0.287645
Prim1	NA	Up	49	2.935345	0.019553	0.340675	0.251913
Nuf2	NA	Up	48	2.974138	0.00309	0.336232	0.275905
Sgol1	NA	Up	48	2.997845	0.002882	0.333573	0.28125
Cenpi	NA	Up	47	2.99569	0.017411	0.333813	0.26534
Kif20a	NA	Up	47	3.0625	0.001547	0.326531	0.30589
Orc1	NA	Up	47	2.989224	0.022176	0.334535	0.25024
Tacc3	NA	Up	46	2.939655	0.025243	0.340176	0.287225
H2afz	NA	Up	45	3.002155	0.040379	0.333094	0.188816
Cep55	NA	Up	44	3.071121	0.000625	0.325614	0.323656
Cks2	NA	Up	43	2.980603	0.001636	0.335503	0.301077
Lrr1	NA	Up	43	3.047414	0.025254	0.328147	0.28961
Spag5	NA	Up	42	3.096983	0.000598	0.322895	0.327866
Arhgap11a	NA	Up	41	3.090517	0.043119	0.32357	0.316706

**Table 5:** The topological characteristics of the top 30 nodes in the PPI network of EC-induced NSPC genetic alterations. NA means not overlapped between the EC-PPI and NSPC-PPI networks.

Gene	Overlap	Regulate	Degree	Average shortest path length	Betweenness centrality	Closeness centrality	Topological coefficient
Gapdh	NA	Down	129	2.303797	0.21952	0.434066	0.040603
Fn1	NA	Down	83	2.586881	0.062983	0.386566	0.054676
Vegfa	NA	Down	64	2.638665	0.041079	0.37898	0.063087
Decr1	NA	Down	59	2.609896	0.066189	0.383157	0.059219
Cd44	NA	Down	57	2.693901	0.029026	0.371209	0.069522
Ccnd1	NA	Down	54	2.703107	0.040282	0.369945	0.065015
Gfap	NA	Down	51	2.663982	0.042334	0.375378	0.070175
Gad1	NA	Up	47	2.837745	0.023163	0.352393	0.075753
Cxcr4	NA	Down	45	2.805524	0.015376	0.35644	0.091761
Pkm	NA	Down	45	2.780207	0.042856	0.359685	0.072881
Anxa5	NA	Down	41	2.817031	0.007322	0.354984	0.091351
Timp1	NA	Down	41	2.805524	0.010114	0.35644	0.09083
Agt	NA	Down	40	2.857307	0.014755	0.34998	0.0945
Cav1	NA	Down	40	2.788262	0.021742	0.358646	0.081866
H6pd	Overlap	Down	39	2.905639	0.018002	0.344158	0.081197
Calb1	NA	Up	39	2.827388	0.022978	0.353683	0.082931
Ctgf	NA	Down	38	2.79977	0.019484	0.357172	0.08851
Pdgfrb	NA	Down	38	2.840046	0.010704	0.352107	0.092062
Snap25	NA	Up	38	2.840046	0.026457	0.352107	0.071554
Fos	NA	Down	37	2.757192	0.026678	0.362688	0.086654
Gad2	NA	Up	37	2.842348	0.016537	0.351822	0.0868
Anxa1	NA	Down	36	2.912543	0.010209	0.343343	0.091158
Tpi1	NA	Down	36	2.905639	0.015808	0.344158	0.086608
Gria1	NA	Up	35	2.837745	0.033782	0.352393	0.079529
Ldha	NA	Down	35	2.933257	0.010636	0.340918	0.096276
Thy1	NA	Down	35	2.886076	0.018426	0.346491	0.090796
Tubb3	NA	Up	35	2.829689	0.013785	0.353396	0.087982
Npy	NA	Up	34	2.906789	0.010344	0.344022	0.099831
Sst	NA	Up	34	2.921749	0.012787	0.342261	0.101852
Gng3	NA	Up	32	3.179517	0.007673	0.314513	0.107118

**Table 6:** The topological characteristics of overlapped DEGs in the PPI network of NSPC-induced EC genetic alterations. The overlapped DEGs mean these DEGs were overlapped between the PPI network of NSPC-induced EC alteration and the PPI network of EC-induced NSPC alteration.

Overlapped DEGs	Regulate	Degree	Average shortest path length	Betweenness centrality	Closeness centrality	Topological coefficient
Timp3	Down	21	3.394397	0.012355	0.294603	0.193591
Mmp14	Down	15	3.321121	0.006668	0.301103	0.213333
Col11a1	Down	14	3.681034	0.000966	0.271663	0.265873
H3f3b	Up	13	3.793103	3.40*E* − 05	0.263636	0.329522
Loxl1	Down	13	3.568966	0.001462	0.280193	0.254605
Ccp110	Up	12	3.497845	0.007609	0.28589	0.324906
Rpl30	Up	12	3.715517	0.001937	0.269142	0.208333
Irs1	Down	11	3.103448	0.01987	0.322222	0.171937
Aldh1a1	Down	9	3.403017	0.022894	0.293857	0.139918
P4ha2	Down	9	3.913793	0.00226	0.255507	0.381313
Cck	Up	8	3.579741	0.011327	0.27935	0.194444
Plod2	Down	8	4.118534	7.55*E* − 06	0.242805	0.428977
Rhoq	Down	8	4.008621	0.012817	0.249462	0.156863
H6pd	Down	7	3.534483	0.017785	0.282927	0.159664
Luc7l3	Up	7	3.571121	0.009536	0.280024	0.188065
Dlx5	Up	6	3.784483	0.007836	0.264237	0.215278
Rcn3	Down	6	3.525862	0.008417	0.283619	0.330247
F3	Down	5	3.56681	7.31*E* − 06	0.280363	0.413514
Hspa2	Down	5	3.221983	0.000827	0.310368	0.275229
Tgm2	Down	5	3.538793	0.005005	0.282582	0.379221
Aldh1a7	Down	4	4.165948	0.000317	0.240041	0.416667
Irgm2	Down	4	4.290948	0.000557	0.233049	0.3125
Smad6	Down	4	3.928879	0.002692	0.254526	0.289474
Bmper	Down	3	4.810345	0.000753	0.207885	0.433333
Daam2	Down	3	3.87931	0.008654	0.257778	0.333333
Rras	Down	3	4.3125	0.000343	0.231884	0.439394
Syde1	Down	3	4.939655	0.000344	0.202443	0.545455
Tns1	Down	3	4.286638	0.002258	0.233283	0.355556
Fcgrt	Down	2	4.157328	0	0.240539	0.576923
Rin2	Down	2	4.681034	0.000218	0.213628	0.5
Sdpr	Down	2	4.275862	0.000187	0.233871	0.5
Slc2a10	Down	2	4.176724	0	0.239422	0.738095

**Table 7:** The topological characteristics of overlapped DEGs in the PPI network of EC-induced NSPC genetic alterations. The overlapped DEGs mean these DEGs were overlapped between the PPI network of NSPC-induced EC alteration and the PPI network of EC-induced NSPC alteration.

Overlapped DEGs	Regulate	Degree	Average shortest path length	Betweenness centrality	Closeness centrality	Topological coefficient
H6pd	Down	39	2.905639	0.018002	0.344158	0.081197
Mmp2	Down	30	2.905639	0.009375	0.344158	0.111791
Cck	Up	24	3.161105	0.002392	0.316345	0.14388
Irs1	Down	24	2.90794	0.011375	0.343886	0.090661
Igfbp5	Down	18	3.002302	0.001391	0.333078	0.145524
Loxl1	Down	18	3.331415	0.002588	0.300173	0.154147
Mmp14	Down	18	2.958573	0.006212	0.338001	0.147655
Aldh1a1	Down	16	3.079402	0.008487	0.324738	0.124419
Plod2	Down	16	3.535098	0.002197	0.282878	0.168269
Col11a1	Down	15	3.464902	0.007219	0.288608	0.154386
Timp3	Down	15	3.065593	0.004764	0.326201	0.152648
Irgm2	Down	14	3.910242	4.82*E* − 05	0.255739	0.348397
P4ha2	Down	14	3.637514	0.001633	0.274913	0.177721
Rhoq	Down	13	3.673188	0.001094	0.272243	0.173382
F3	Down	11	3.150748	0.00066	0.317385	0.188776
Dlx5	Up	10	3.500575	0.001701	0.285667	0.221359
Hspa2	Down	10	3.356732	0.009675	0.297909	0.113084
Luc7l3	Up	9	3.771001	0.004972	0.265182	0.17284
Syde1	Down	9	3.562716	0.005367	0.280685	0.202822
Tgm2	Down	9	3.103567	0.002864	0.32221	0.194714
Rras	Down	8	3.651323	0.00461	0.273873	0.227113
Sdpr	Down	8	3.372842	0.002511	0.296486	0.180118
Aldh1a7	Down	7	3.686997	0.000587	0.271223	0.221254
Ccp110	Up	7	3.982739	0.002565	0.251084	0.159664
Cdc7	Up	7	3.821634	0.000152	0.261668	0.313589
Rcn3	Down	7	3.697353	0.000432	0.270464	0.249433
Tns1	Down	7	3.397008	0.005802	0.294377	0.188729
Zcchc12	Up	6	3.775604	0.006142	0.264858	0.217391
Akap12	Down	5	3.422325	0.002599	0.292199	0.261111
Daam2	Down	5	3.658228	0.000694	0.273356	0.303226
H3f3b	Up	5	4.362486	6.29*E* − 05	0.229227	0.5125
Rpl30	Up	5	4.084005	0.000687	0.244858	0.3
Sema3c	Down	5	3.528193	0.002593	0.283431	0.2075
Smad6	Down	5	3.510932	0.001004	0.284825	0.284848
Bmper	Down	4	4.210587	0.00075	0.237497	0.26
Rin2	Down	4	3.874568	0.002452	0.258093	0.25
Gem	Down	3	3.882624	0.002302	0.257558	0.42735
Selenbp1	Down	3	4.462601	0.000293	0.224085	0.458333
Fcgrt	Down	2	3.943613	3.45*E* − 05	0.253575	0.5
Slc2a10	Down	2	3.864212	6.35*E* − 05	0.258785	0.5
Sytl2	Down	2	3.759494	0.000207	0.265993	0.5
Fam102a	Down	1	5.730725	0	0.174498	0
Macrod1	Down	1	5.880322	0	0.170059	0
Pdgfrl	Down	1	4.286536	0	0.233289	0

**Table 8:** The topological characteristics of the top 30 nodes in the TF-target DEG interaction network involved in the NSPC-induced EC alteration.

Name	Label	Degree	Average shortest path length	Betweenness centrality	Closeness centrality	Topological coefficient
Ptgs2	Upregulated DEG	30	3.17073171	0.25370241	0.31538462	0.07348485
Il6	Upregulated DEG	20	3.31300813	0.16071429	0.30184049	0.10921053
Serpine1	Upregulated DEG	19	3.40650407	0.10638454	0.29355609	0.09078947
Mmp9	Downregulated DEG	18	3.25203252	0.16761464	0.3075	0.11243386
Sp1	TF	15	3.17479675	0.15105709	0.31498079	0.10813008
Nfkb1	TF	14	2.99593496	0.17606132	0.33378562	0.11316212
Mmp2	Downregulated DEG	13	3.45121951	0.05861543	0.28975265	0.16008316
Fgf10	Downregulated DEG	13	4.18699187	0.10945704	0.23883495	0.07692308
Trp53	TF	13	3.42276423	0.10326907	0.29216152	0.10697115
Jun	TF	13	3.14634146	0.14436348	0.31782946	0.12725546
Twist1	Downregulated DEG_TF	12	3.50406504	0.13470851	0.28538283	0.13288288
Col1a1	Downregulated DEG	12	3.4796748	0.11936909	0.28738318	0.13709677
Birc5	Upregulated DEG	11	3.53252033	0.1098108	0.283084	0.13636364
Csf2	Upregulated DEG	10	3.51626016	0.08268292	0.28439306	0.16071429
Smad6	Downregulated DEG	9	4.94715447	0.04322026	0.2021364	0.12698413
Stat3	TF	9	3.43495935	0.09055949	0.29112426	0.15065913
Egr1	TF	9	3.76829268	0.05361397	0.26537217	0.13947991
Nfe2l2	Upregulated DEG_TF	8	3.89430894	0.03472983	0.25678497	0.15972222
Rela	TF	8	3.33333333	0.05841757	0.3	0.18382353
Ep300	TF	8	3.52439024	0.04168611	0.28373702	0.19067797
Upp1	Upregulated DEG	7	3.58536585	0.04680464	0.27891156	0.1978022
Igf2	Downregulated DEG	7	5.18292683	0.04071495	0.19294118	0.14285714
Ebf1	Downregulated DEG_TF	7	4.57723577	0.04361263	0.21847247	0.14285714
Fos	TF	6	3.82926829	0.01974273	0.2611465	0.22727273
Dcstamp	Upregulated DEG	6	3.75203252	0.02000123	0.26652221	0.25396825
Nqo1	Upregulated DEG	5	3.83333333	0.02317522	0.26086957	0.21904762
Ndrg1	Downregulated DEG	5	3.83333333	0.02104419	0.26086957	0.27142857
Mmp14	Downregulated DEG	5	3.81707317	0.01378142	0.26198083	0.29
Maf	Downregulated DEG_TF	5	3.84146341	0.0340471	0.26031746	0.28571429
Hspa1b	Downregulated DEG	5	3.88211382	0.05662735	0.25759162	0.24545455

**Table 9:** The topological characteristics of the top 30 nodes in the TF-target DEG interaction network involved in the EC-induced NSPC alteration.

Name	Label	Degree	Average shortest path length	Betweenness centrality	Closeness centrality	Topological coefficient
Cdkn1a	Downregulated DEG	65	2.73913043	0.30858346	0.36507937	0.0283698
Ccnd1	Downregulated DEG_TF	44	2.90096618	0.18721585	0.34471274	0.0390556
Sp1	TF	38	2.75120773	0.18665254	0.36347673	0.04086687
Fos	Downregulated DEG_TF	37	2.89855072	0.15489676	0.345	0.05012705
Vegfa	Downregulated DEG	24	3.09178744	0.1032236	0.3234375	0.06904762
Hes1	Downregulated DEG_TF	21	3.26328502	0.093159	0.30643967	0.06776557
Cdkn2a	Downregulated DEG	20	3.16183575	0.06806015	0.31627196	0.0622093
Nfkb1	TF	19	2.83091787	0.10896078	0.35324232	0.078356
Cebpb	Downregulated DEG_TF	15	3.01690821	0.0630859	0.33146517	0.07979003
Acta2	Downregulated DEG	14	3.41062802	0.04841393	0.29320113	0.10449735
Trp53	TF	14	3.23188406	0.04815141	0.30941704	0.09895151
Jun	TF	14	3.25603865	0.03607564	0.30712166	0.10204082
Mmp2	Downregulated DEG	13	3.08937198	0.03126342	0.32369038	0.11609687
Ep300	TF	12	3.13043478	0.05353532	0.31944444	0.11131841
Cdkn2b	Downregulated DEG	12	3.80917874	0.03041314	0.26252378	0.10714286
Nfe2l2	Downregulated DEG_TF	12	3.26570048	0.04141463	0.30621302	0.10585586
Rela	TF	11	3.21497585	0.03799736	0.31104433	0.118411
Nr3c1	Downregulated DEG_TF	11	3.24637681	0.03066868	0.30803571	0.11162255
Smad6	Downregulated DEG_TF	11	3.73671498	0.02413405	0.26761474	0.11004785
Sp3	TF	10	3.35990338	0.01668852	0.29762761	0.12268041
Stat3	TF	10	3.33816425	0.03841092	0.29956585	0.13235294
Id3	Downregulated DEG	10	3.82125604	0.02673839	0.26169406	0.10869565
Smad3	TF	9	3.72463768	0.03330304	0.26848249	0.14403292
Egr1	TF	9	3.57004831	0.03896833	0.28010825	0.12007168
Mbp	Downregulated DEG	9	3.52415459	0.02687184	0.283756	0.13888889
Srebf1	TF	8	3.59903382	0.0307691	0.27785235	0.13380282
Myc	TF	8	3.36231884	0.02336862	0.29741379	0.14650538
Id1	Downregulated DEG_TF	8	3.2826087	0.02185908	0.30463576	0.13903061
Cebpd	Downregulated DEG	8	3.43236715	0.01679448	0.29134412	0.16163793
Pdk4	Downregulated DEG	8	3.61111111	0.01282401	0.27692308	0.19396552

**Table 10:** The number of experimentally validated, computationally predicted, and total miRNA-target DEG interaction pairs during the NSPC-induced EC alteration and EC-induced NSPC alteration.

Coculture-induced alterations in one type of cells	Validated miRNA-target DEG pairs	Predicted miRNA-target DEG pairs	Total miRNA-target DEG pairs (including validated and predicted)	Overlapped miRNA-target DEG interaction pairs (overlapped between NSPC-induced EC alteration and EC-induced NSPC alteration)
NSPC-induced EC alteration	536	391	810	The overlapped 14 interaction pairs are as follows: miR-21-Rhoq, miR-23a-Akap12, miR-23a-Aldh1a1, miR-23a-Exoc3l4, miR-23a-Gm5424, miR-23a-Mfhas1, miR-23a-Nfe2l2, miR-23a-Slc16a6, miR-23b-Akap12, miR-23b-Aldh1a1, miR-23b-Gm5424, miR-23b-Mfhas1, miR-23b-Nfe2l2, and miR-23b-Slc16a6.
EC-induced NSPC alteration	571	243	711	

**Table 11:** The topological characteristics of the top 30 nodes in the DEmiRNA-target network involved in the NSPC-induced EC alteration.

Name	lab2	Degree	Average shortest path length	Betweenness centrality	Closeness centrality	Topological coefficient
mmu-miR-26a-2	DEmiRNA	2371	2.195604	0.216585	0.455455	0.221505
mmu-miR-26a-1	DEmiRNA	2371	2.195604	0.216585	0.455455	0.221505
mmu-let-7i	DEmiRNA	2244	2.238546	0.131513	0.446719	0.323641
mmu-let-7c-2	DEmiRNA	2202	2.252747	0.110037	0.443902	0.332539
mmu-let-7c-1	DEmiRNA	2202	2.252747	0.110037	0.443902	0.332539
mmu-let-7a-2	DEmiRNA	1585	2.461369	0.051855	0.406278	0.376735
mmu-let-7a-1	DEmiRNA	1585	2.461369	0.051855	0.406278	0.376735
mmu-miR-23a	DEmiRNA	1297	2.558749	0.106936	0.390816	0.247494
mmu-miR-23b	DEmiRNA	1269	2.568216	0.100185	0.389375	0.248916
mmu-let-7d	DEmiRNA	1252	2.573965	0.063356	0.388506	0.353484
mmu-miR-199a-2	DEmiRNA	839	2.713609	0.055721	0.368513	0.273838
mmu-miR-199a-1	DEmiRNA	839	2.713609	0.055721	0.368513	0.273838
mmu-miR-140	DEmiRNA	760	2.740321	0.114496	0.364921	0.175411
mmu-miR-182	DEmiRNA	668	2.771429	0.088383	0.360825	0.194891
mmu-miR-199b	DEmiRNA	660	2.774134	0.048537	0.360473	0.278883
mmu-miR-210	DEmiRNA	468	2.839053	0.061318	0.35223	0.195913
mmu-miR-99b	DEmiRNA	52	2.979713	0.006853	0.335603	0.234375
Celf1	Target gene	16	2.004227	0.002733	0.498946	0.24026
Taok1	Target gene	15	2.179374	0.001948	0.458847	0.263894
Ubn2	Target gene	14	2.137447	0.001613	0.467848	0.279439
Suco	Target gene	14	2.137447	0.001613	0.467848	0.279439
Mtf2	Target gene	14	2.186475	0.001497	0.457357	0.28312
Map3k2	Target gene	14	2.162468	0.001485	0.462435	0.282045
Kdm6a	Target gene	14	2.162468	0.001485	0.462435	0.282045
Ino80d	Target gene	14	2.137109	0.001824	0.467922	0.267984
Celf2	Target gene	14	2.090786	0.001937	0.478289	0.265165
Ankrd44	Target gene	14	2.186475	0.001497	0.457357	0.28312
Tnpo1	Target gene	13	2.196281	0.001334	0.455315	0.291288
Rfx7	Target gene	13	2.144548	0.001367	0.466299	0.28419
Kmt2a	Target gene	13	2.263567	0.001442	0.441781	0.289898

**Table 12:** The topological characteristics of the top 30 nodes in the DEmiRNA-target network involved in the EC-induced NSPC alteration.

Name	lab2	Degree	Average shortest path length	Betweenness centrality	Closeness centrality	Topological coefficient
mmu-let-7b	DEmiRNA_up	2362	2.046362	0.551173	0.488672	0.170618
mmu-miR-124-1	DEmiRNA_down	1863	2.247531	0.154513	0.444933	0.394448
mmu-miR-124-2	DEmiRNA_down	1863	2.247531	0.154513	0.444933	0.394448
mmu-miR-124-3	DEmiRNA_down	1863	2.247531	0.154513	0.444933	0.394448
mmu-miR-23a	DEmiRNA_up	1297	2.475711	0.158061	0.403924	0.306091
mmu-miR-23b	DEmiRNA_up	1269	2.486999	0.14813	0.402091	0.309242
mmu-miR-320	DEmiRNA_up	690	2.720419	0.141572	0.36759	0.217805
mmu-miR-210	DEmiRNA_up	468	2.809917	0.093463	0.355882	0.206654
Ino80d	Target gene	8	1.998387	0.0024	0.500403	0.294443
Adam10	Target gene	8	1.998387	0.0024	0.500403	0.294443
Mtf2	Target gene	8	1.998387	0.0024	0.500403	0.294443
Pcdh19	DEmRNA	7	2.085467	0.001524	0.479509	0.337695
Rora	DEmRNA	7	2.545051	0.001403	0.392919	0.369491
Ago2	Target gene	7	2.1282	0.00156	0.469881	0.338576
Ago3	Target gene	7	2.1282	0.00156	0.469881	0.338576
Col4a1	Target gene	7	2.085467	0.001524	0.479509	0.337695
Cpd	Target gene	7	2.085467	0.001524	0.479509	0.337695
Map3k1	Target gene	7	2.085467	0.001524	0.479509	0.337695
Mecp2	Target gene	7	2.085467	0.001524	0.479509	0.337695
Etnk1	Target gene	7	2.085467	0.001524	0.479509	0.337695
Kpna1	Target gene	7	2.085467	0.001524	0.479509	0.337695
Yod1	Target gene	7	2.085467	0.001524	0.479509	0.337695
Prtg	Target gene	7	2.085467	0.001524	0.479509	0.337695
Slc36a1	Target gene	7	2.1282	0.00156	0.469881	0.338576
Tet2	Target gene	7	2.1282	0.00156	0.469881	0.338576
Ahctf1	Target gene	7	2.1282	0.00156	0.469881	0.338576
Qser1	Target gene	7	2.085467	0.001524	0.479509	0.337695
Wnk1	Target gene	7	2.085467	0.001524	0.479509	0.337695
Slc7a2	Target gene	7	2.085467	0.001524	0.479509	0.337695
Aldh1l2	Target gene	7	2.545051	0.001403	0.392919	0.369491

**Table 13:** The 16 small molecular drugs that were overlapped between NSPC-induced EC genetic alteration and EC-induced NSPC genetic alteration.

Small molecular drugs	Enrichment in the NSPC-induced EC alteration	Enrichment in the EC-induced NSPC alteration
2-Aminobenzenesulfonamide	0.629	-0.682
Adiphenine	0.748	-0.693
Deptropine	-0.677	-0.672
Diphenhydramine	0.67	-0.573
Felbinac	0.719	-0.697
Fludrocortisone	0.503	-0.496
Fluphenazine	-0.357	-0.378
Ikarugamycin	0.811	-0.838
Lycorine	0.633	0.776
Milrinone	-0.779	0.73
PHA-00745360	0.567	-0.514
Podophyllotoxin	0.661	-0.933
Quinostatin	-0.992	-0.954
Resveratrol	-0.479	-0.504
Thiamazole	0.726	0.543
Triflupromazine	0.694	-0.71

**Table 14:** The scores of the crosstalk miRNA interaction pairs under five conditions including the target genes, small molecular drugs, chemical compounds, signaling pathways, and biological processes. These crosstalk miRNAs link the EC-induced NSPC alteration process and NSPC-induced EC alteration process. The scores were calculated by the “Meet/Min” formula.

miRNAs involved in the NSPC-induced EC alteration	miRNAs involved in the EC-induced NSPC alteration	Score_target	Score_molecular	Score_chemical	Score_pathway	Score_bp
mmu-miR-210	mmu-miR-210	1	0.166667	1	1	1
mmu-miR-23b	mmu-miR-23b	1	0.075	1	1	1
mmu-miR-23b	mmu-miR-23a	0.966903	0.022222	1	0.946667	0.931284
mmu-miR-23a	mmu-miR-23b	0.966903	0.05	0.95	0.946667	0.931284
mmu-let-7d	mmu-let-7b	0.834665	0.021739	0.2	1	0.862256
mmu-miR-26a-1	mmu-miR-23a	0.393215	0.05	0.321429	0.936709	0.614696
mmu-miR-26a-2	mmu-miR-23a	0.393215	0.05	0.321429	0.936709	0.614696
mmu-miR-26a-1	mmu-miR-23b	0.389283	0.025	0.307692	0.946667	0.615335
mmu-miR-26a-2	mmu-miR-23b	0.389283	0.025	0.307692	0.946667	0.615335
mmu-miR-26a-1	mmu-miR-210	0.344017	0.027778	0.333333	0.8	0.689956
mmu-miR-26a-2	mmu-miR-210	0.344017	0.027778	0.333333	0.8	0.689956
mmu-miR-26a-1	mmu-miR-320	0.337681	0.05	0.4	0.885714	0.708661
mmu-miR-26a-2	mmu-miR-320	0.337681	0.05	0.4	0.885714	0.708661
mmu-miR-23a	mmu-let-7b	0.312259	0.021739	0.25	0.772152	0.595539
mmu-let-7i	mmu-miR-23b	0.3026	0.074074	0.318182	0.693333	0.592737
mmu-let-7i	mmu-miR-23a	0.302236	0.074074	0.363636	0.708861	0.590143
mmu-miR-199a-1	mmu-let-7b	0.299166	0.022222	0.142857	0.728395	0.65798
mmu-miR-199a-2	mmu-let-7b	0.299166	0.022222	0.142857	0.728395	0.65798
mmu-let-7c-1	mmu-miR-23b	0.29866	0.071429	0.32	0.72	0.587937
mmu-let-7c-2	mmu-miR-23b	0.29866	0.071429	0.32	0.72	0.587937
mmu-let-7i	mmu-miR-210	0.297009	0.037037	0.222222	0.8	0.609898
mmu-miR-210	mmu-let-7b	0.292735	0.073171	0.875	0.8	0.63901
mmu-let-7c-1	mmu-miR-124-1	0.285561	0.035714	0.72	0.709302	0.623492
mmu-let-7c-1	mmu-miR-124-2	0.285561	0.035714	0.72	0.709302	0.623492
mmu-let-7c-1	mmu-miR-124-3	0.285561	0.035714	0.72	0.709302	0.623492
mmu-let-7c-2	mmu-miR-124-1	0.285561	0.035714	0.72	0.709302	0.623492
mmu-let-7c-2	mmu-miR-124-2	0.285561	0.035714	0.72	0.709302	0.623492
mmu-let-7c-2	mmu-miR-124-3	0.285561	0.035714	0.72	0.709302	0.623492
mmu-let-7a-1	mmu-miR-23b	0.239559	0.041667	0.619048	0.706667	0.616536
mmu-let-7a-2	mmu-miR-23b	0.239559	0.041667	0.619048	0.706667	0.616536
mmu-let-7a-1	mmu-miR-23a	0.2367	0.041667	0.666667	0.721519	0.610834
mmu-let-7a-2	mmu-miR-23a	0.2367	0.041667	0.666667	0.721519	0.610834
mmu-miR-23b	mmu-miR-124-1	0.230102	0.028571	0.666667	0.8	0.527677
mmu-miR-23b	mmu-miR-124-2	0.230102	0.028571	0.666667	0.8	0.527677
mmu-miR-23b	mmu-miR-124-3	0.230102	0.028571	0.666667	0.8	0.527677
mmu-miR-23a	mmu-miR-124-1	0.229761	0.028571	0.75	0.759494	0.523207
mmu-miR-23a	mmu-miR-124-2	0.229761	0.028571	0.75	0.759494	0.523207
mmu-miR-23a	mmu-miR-124-3	0.229761	0.028571	0.75	0.759494	0.523207
mmu-miR-23a	mmu-miR-320	0.226087	0.04	0.2	0.714286	0.755906
mmu-miR-23b	mmu-miR-320	0.22029	0.043478	0.2	0.628571	0.76378
mmu-miR-199a-1	mmu-miR-23a	0.212157	0.066667	0.142857	0.594937	0.619707
mmu-miR-199a-2	mmu-miR-23a	0.212157	0.066667	0.142857	0.594937	0.619707
mmu-miR-199a-1	mmu-miR-23b	0.205006	0.05	0.142857	0.626667	0.618078
mmu-miR-199a-2	mmu-miR-23b	0.205006	0.05	0.142857	0.626667	0.618078
mmu-miR-210	mmu-miR-23a	0.202991	0.170732	0.25	0.8	0.660844
mmu-miR-210	mmu-miR-23b	0.196581	0.075	0.125	0.8	0.672489
mmu-let-7d	mmu-miR-23b	0.183706	0.025	0.64	0.666667	0.675705
mmu-let-7d	mmu-miR-23a	0.182109	0.022222	0.64	0.717949	0.671367
mmu-let-7d	mmu-miR-320	0.176812	0.057692	0.2	0.485714	0.531496
mmu-miR-199a-1	mmu-miR-320	0.127536	0.022222	0.2	0.771429	0.616142
mmu-miR-199a-2	mmu-miR-320	0.127536	0.022222	0.2	0.771429	0.616142
mmu-miR-182	mmu-miR-320	0.115269	0.093023	0.666667	0.828571	0.555118
mmu-miR-199b	mmu-miR-320	0.109091	0.090909	0.6	0.714286	0.543307
mmu-miR-210	mmu-miR-320	0.087607	0.121951	0.2	0.2	0.358268

## Data Availability

The data used to support the findings of this study are available from the corresponding author upon request.
